# Financial health and economic growth responsiveness as solution to environmental degradation in Pakistan

**DOI:** 10.1007/s11356-024-33176-2

**Published:** 2024-04-18

**Authors:** Mansoor Ahmed Golo, Dongping Han, Daniel Balsalobre-Lorente, Magdalena Radulescu

**Affiliations:** 1 School of Management, Harbin Institute of Technology, Harbin, People’s Republic of China; 2 Department of Applied Economics I, University Castilla La-Mancha, 13071 Cuenca, Spain; 3 Department of Management and Marketing, Faculty of Economics and Management, Czech University of Life Sciences Prague, Prague, Czech Republic; 4 UNEC Research Methods Application Center, Azerbaijan State University of Economics (UNEC), Istiqlaliyyat Str. 6, Baku, 1001 Azerbaijan; 5 Department of Finance, Accounting and Finance, National University of Science and Technology Politehnica Bucharest, Bucharest, Romania; 6 Institute of Doctoral and Post-Doctoral Studies, University Lucian Blaga of Sibiu, Sibiu, Romania

**Keywords:** Environment, Awareness, Population, Monetary dynamics, ARDL bounds testing, Pakistan

## Abstract

This study analyses the impact of GDP per capita, domestic credit, savings, and population on the environment in Pakistan from 1995 to 2019. The country has consistently been ranked eighth, fifth, and eight as an environmentally vulnerable nation from 1998 to 2017, 1999 to 2018, and 2000 to 2019, respectively. Therefore, the study explores the role of environmental awareness as a potential strategy for making peace with nature. Findings of the ARDL bounds testing approach confirm the long-run cointegration among variables of concern. In further assessment, the study determines that increased per capita income is detrimental to environmental quality in the long run. However, in the short run, it shows a favourable impact. On the other hand, domestic credit worsens the environment in the long and short runs. However, savings are positively insignificant in this regard. Furthermore, the total population significantly harms the environment in the long and short run. Fortunately, environmental awareness has emerged as a key solution to environmental degradation in Pakistan. Findings show that an increase in the dissemination of environmental awareness through the Internet, mobile, and landline phones, as well as by increasing education expenditures, mitigates the populations’ detrimental impact on the environment and improves environmental quality in the long run.

**Graphical Abstract:**

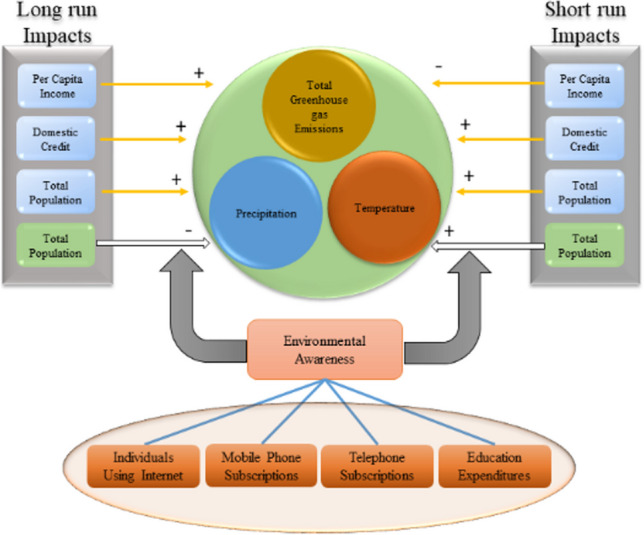

## Introduction

The environment is a natural canvas of our only home; every material we produce, consume, or waste within this confined space lingers and echoes its presence for generations (d’Arge and Kogiku 1973). In the current era of unyielding monetary growth targets and widespread demographic shifts, safeguarding our shared home, the Earth, has increasingly become a challenge because anthropogenic activities such as the utilization of natural, energy, and financial resources for monetary growth have significantly diminished environmental quality by polluting water, soil, and air (Kucheryavyy et al. 2023; Marfatia 2023). According to the United Nations Economic and Social Commission for Western Asia, any undesirable change in natural settings caused by the depletion of air, water, and soil, as well as the destruction of ecosystems and the extinction of wildlife, is called environmental degradation (Singh et al. 2022). Poor ecological quality threatens human security through extreme weather events (Ide et al. 2020). In the near past, the world has witnessed such natural catastrophes, including forest fires, droughts, heatwaves, tornadoes, unpredictable monsoons, rivers, flash, coastal floods, etc. (Jegasothy et al. 2023; Zhou et al. 2023).,^1^, ^2^ The resulting impacts of such incidents can be observed across socioeconomic dimensions, including water scarcity, the spread of diseases, and biodiversity loss (Akbar et al. 2021; Dong et al. 2021).

**Fig. 1:**
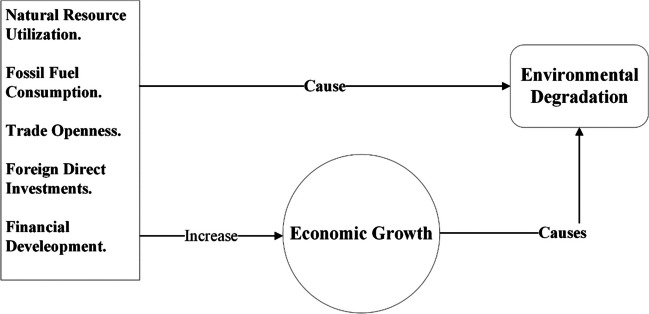
Monetary aspects and environmental degradation (theorized by authors)

**Fig. 2:**
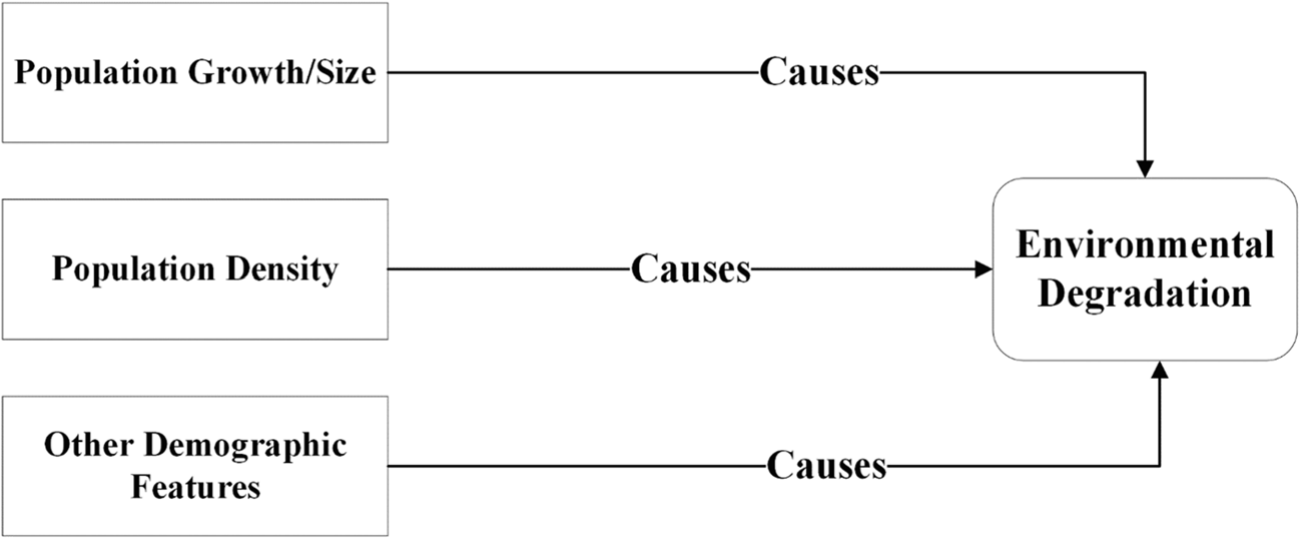
Demographic causes of environmental degradation (theorized by authors)

**Fig. 3:**
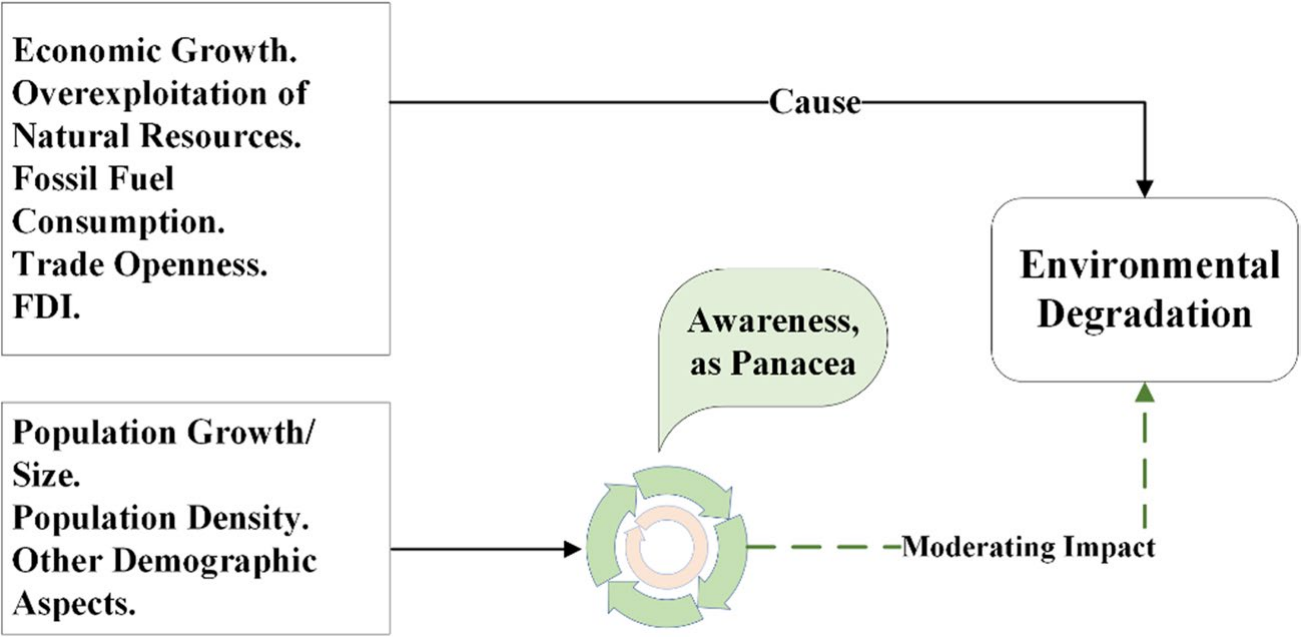
Environmental awareness as panacea (theorized by authors)

Additionally, food and water contamination and decreasing crop yields contribute more to the existing challenges.^3^ A persistent decline in crop productivity may lead to food insecurity, hindering the achievement of Sustainable Development Goal 2, which focuses on zero hunger worldwide.^4^ Environmental degradation is an existential threat to humans on Earth (Ekoh et al. 2023). Due to the underlying concerns, the importance of natural settings is gaining global attention (Li et al. 2023). People from every walk of life call for action to stop the “apocalyptic” impact of the worsening environment, which underscores the need for a coherent response to curb and mitigate the causes of environmental degradation. The situation provides countries with a basis for climate action (Peisker 2023). Given that, the current study not only uncovers monetary and demographic causes of environmental degradation but also empirically examines the existing fundamentals of acquiring knowledge as a panacea to the problem (Figs. 1, 2, and 3).

Concerning the causes of environmental degradation, numerous studies found that demographic factors, such as population size and density, as well as urbanization, could harm the environment (Dogan and Turkekul 2016). As the number of individuals grows, they encroach on natural settlements, which cause a disturbance in ecosystems (Rajak et al. 2023). The growing population also increases land utilization for crop production, resulting in soil erosion and causing land degradation (Yousaf et al. 2022). Besides that, monetary growth also contributes to environmental degradation. Because growth-driven activities, including industrial output, trade, tourism, etc., often overexploit natural and energy resources, which results in a poor environment due to industrial waste and hazardous emissions (Adebayo et al. 2023; Koengkan et al. 2023), like other developing countries, in Pakistan, growth-associated activities and population dynamics carry environmental risks (Jafri et al. 2023). Pakistan is an environmentally susceptible nation and the fifth largest population on Earth.^5^ The country is also ranked fifth as the most climatically vulnerable in the Global Climate Risk Index Report 2018.^6^ The World Bank’s report “South Asia’s Hotspots” underscored Pakistan’s climatic vulnerability (Mani et al. 2018). Besides that, the National Climate Change Policy of Pakistan, adopted in 2012, also acknowledges the country’s environmental vulnerability.^7^

As António Guterres passionately reminds us (Guterres 2020),^8^ humanity is in a dangerous clash with nature, and our current path is nothing less than self-destruction. Biodiversity and ecosystems are collapsing, and the chaos we sow comes back to haunt us with ever-increasing intensity. However, there is a ray of hope; human actions can navigate us away from disastrous situations. In this context, our study serves as a beacon of hope, unveiling groundbreaking solutions that enable Pakistan to make peace with nature. To achieve this, the current study transforms human actions by inculcating environmental awareness into Sustainable Development Goal Thirteen (SDG 13). Pakistan has fifth largest population, suggesting that the country has a demographic potential to contribute positively to environmental preservation. The National Assembly of Pakistan has already passed a Climate Action Bill, which aligns with international conventions on climate change. In Chapter Three, Article 8, the bill outlines the functions of the Climate Action Authority as empowered by the legislation.

The chapter defines guidelines for exercising the authority’s powers to mitigate environmental degradation. In this regard, sub-articles (o), (p), (s), and (t) of Article 8 within Chapter Three precisely task the authorities with designing, planning, and executing environmental awareness initiatives among the citizens. However, there remains ambiguity regarding the methods for disseminating awareness related to environmental concerns. Besides that, previous studies have examined the impact of monetary and demographic variables on the environment in Pakistan. Still, the aspects this study uncovers have not been considered in the available literature. For example, previous studies have taken CO_2_ emissions, total greenhouse gas emissions, and ecological footprints as individual measures of environmental degradation. None of the available studies focuses on the composite index of environmental measures, which this study considers. The study incorporates total greenhouse gas emissions, temperature, and precipitation as an environmental degradation index.

Moreover, by filling the policy gap, the findings of this study offer innovative solutions that can assist policymakers in gradually removing Pakistan from the list of environmentally vulnerable nations. For this purpose, the current study estimates a composite index of environmental awareness, which comprises individuals using the Internet, mobile and fixed-line phone subscriptions, and the government’s expenditure on education. The empirically tested methods of disseminating environmental awareness by this study not only extend the latest information into the available literature but also mathematically support the sub-articles 8(o), 8(p), 8(s), and 8(t) of the Pakistan Climate Change Act (2017). Therefore, to address the identified gaps, this study endeavours to formulate the following research questions:

1. How do monetary dynamics affect environmental excellence, and which aspects of economic development harm the environment?
2. Whether and how population aspects influence natural settings.
3. Whether and how the fundamentals of acquiring knowledge can help mitigate environmental degradation.

The attainable objectives are set below.

1. To empirically analyse the impact of per capita income, domestic credit to the private sector, and gross domestic savings on environmental degradation.
2. To investigate the impact of the total population on environmental degradation.
3. To investigate the moderating impact of environmental awareness as a panacea to environmental degradation.

## Literature review

### Environment in Pakistan

Environmental quality in Pakistan is getting toxic day by day (Butt et al. 2022). The causes of environmental degradation in Pakistan are similar to those of other developing countries, such as overexploitation of natural, energy, and financial resources and a significant population sprawl (Ashraf 2023). A study from 1990 to 2017 held that monetary development, energy use, and trade openness are responsible for CO_2_ emissions (Usman et al. 2022). Rapid economic growth and investments in traditional production methods are also accountable for ecological collapse (Li et al. 2023). Moreover, on the one hand, foreign direct investment (FDI) boosts economic growth (Dogan 2013), while on the other hand, it contributes to environmental degradation (Liu et al. 2022a). A study from 1973 to 2017 found a causal relationship between carbon emissions and nuclear energy in Pakistan (Mahmood et al. 2020). Fossil fuel energy is also among the country’s major causes of environmental degradation (Xiuhui and Raza 2022). Besides that, transport, agriculture, and population aspects significantly influence the environment in Pakistan (Koçak et al. 2023; Uzair Ali et al. 2022). A SAARC countries study found population size to be the leading cause of environmental degradation (Rani et al. 2023). In a study from 1971 to 2014, population density was exposed as the primary cause of hazardous emissions (Uzair Ali et al. 2022). Meanwhile, in Pakistan, from 1981 to 2016, a study summarized that population density does not contribute to environmental degradation (Hussain et al. 2022).

Furthermore, in Pakistan, the intensification of air pollutants is attributed to the burning of agricultural waste, fossil fuels, and industrial and traffic emissions (Moyebi et al. 2023) that cause severe respiratory and heart diseases among individuals of all ages in the country (Shakil et al. 2023). Environmental causes of natural calamities put Pakistan in the top 10 list of environmentally vulnerable countries (Zaman 2023). Pakistan has experienced record-breaking precipitation that flooded the Indus River and caused a megaflood in 2010 (Otto et al. 2023). Again, in 2022, a flood brutally hit Pakistan, and the estimated damage surpassed 14 billion dollars. Sindh Province was among the most severely affected regions in Pakistan. A total of 33 million people were directly affected, and more than 15 hundred lost their lives (Government of Pakistan 2021). Pakistan often has the highest trending temperatures, and usually, the country experiences hell-hot weather (M. T. Khan and Imran 2023).

### Monetary aspects and environmental degradation

Due to the changing landscapes, the relationship between economic growth and the environment has become more complex. Because the aspects that help countries grow fiscally have a detrimental impact on environmental quality. For instance, energy use, trade, and tourism may foster economic growth in some countries but have damaging influences on the environment (Dogan et al. 2015). A study from 1971 to 2013 found trade to be the primary culprit behind CO_2_ emissions (Ansari et al. 2020). In addition, the environment is also harmed by income, energy consumption, gross capital formation, foreign direct investment, and gross domestic output (Liu et al. 2022b). A European Union study from 1980 to 2012 revealed that non-renewable energy sources are among the significant determinants of environmental degradation. The results showed that increasing non-renewable energy consumption upsurges carbon emissions. The causality results showed that growing income and trade led to an escalation in CO_2_ emissions (Dogan and Seker 2016b).

Similarly, in an OECD study, results revealed that increasing real income and energy consumption cause hazardous emissions (Dogan and Seker 2016a). However, the direction and magnitude of the impacts in all the underlined studies depend on methodological differences and the countries’ development categories (H. Khan et al. 2021). A study conducted in China from 1988 to 2021 found that increasing real GDP harms environmental degradation. Ecological footprints were taken as environmental measures in the study (Li et al. 2023). Nations often rely on increased industrial production to accomplish fiscal progress, which leads to excessive use of natural, energy, and financial resources and causes hazardous emissions (Ozcan et al. 2020). Similarly, offering loans on easy terms to boost economic growth degrades the environment (Shen et al. 2021). If countries provide credit on soft terms for environment-friendly projects, this could improve environmental quality (Gokmenoglu et al. 2021; Hodžić et al. 2023; Madaleno et al. 2022).

On the other hand, energy-induced economic growth through easy access to finances can lead to environmental collapse. A global study from 1990 to 2017 revealed that energy-induced economic growth positively affects carbon emissions (Zeqiraj et al. 2020). Fossil fuel consumption was found detrimental to the environment in India, Pakistan, and Bangladesh for the period 1971–2014 (Uzair Ali et al. 2022).

### Demographic aspects and environmental degradation

Population aspects such as size and density of the populations, as well as family and age structures, and urbanization, significantly influence the environment (Dogan and Turkekul 2016; Uzair Ali et al. 2022). As populations grow correspondingly, the encroachment on neighbouring natural habitats also rises, resulting in the loss of biodiversity and disruption of the overall functionality of the ecosystem (Zou et al. 2022). In Brazil, a vast biocapacity decline was observed from 1961 to 2016 due to a rapid increase in urban settlements and monetary growth (Ahmed et al. 2022). A SAARC countries study found population size as a leading cause of carbon intensification (Rani et al. 2023). A South Asian study found an increasing number of individuals harmful to the environment from 1995 to 2020 (Amin et al. 2023). In a study conducted in India, Pakistan, and Bangladesh from 1971 to 2014, population density was exposed as the primary cause of hazardous emissions (Uzair Ali et al. 2022). Population density significantly increases environmental pollution, according to a study of seventy countries reported from 2000 to 2017 (Liu et al. 2022a).

Furthermore, a Middle East and North African study from 1991 to 2019 revealed that rapid urban growth is a significant cause of environmental degradation (Sun et al. 2022). A study conducted in China from 1997 to 2018 showed that increased urbanization could intensify CO_2_ emissions (Cheng and Hu 2023). The discussed studies indicate that the total number of individuals, population density, and rising urban settlements could harm the environment. Nevertheless, Wang et al. (2022) found diverse impacts of populations on the environment while examining a global sample of 125 countries from 1990 to 2017. Khan and Imran (2023), in a study from 1990 to 2021, found that population density can reduce CO_2_ emissions in Europe and Central Asia. A study from 1981 to 2016 summarized that population density does not contribute to environmental degradation in Pakistan (Hussain et al. 2022). Though the existing studies reported mixed outcomes, the overall literature demonstrates that the population features shape the environments. The current study also emphasizes the significance of populations in determining the environment.

### Awareness aspects and environmental degradation

There is a valid concern that traditional physical planning efforts may not be sufficient for climate action, as they involve a lengthy time frame and implementation uncertainty (Ren et al. 2023). Hence, along with existing strategies, keeping people aware can be an excellent strategy to promote peace with nature (Dogan 2017; Ferreira et al. 2023). Building a sense of responsibility toward Mother Nature among the population is decisive at this stage (Chen et al. 2023). Decades back in 1972, the UN General Assembly considered the importance of environmental awareness and designated June 5 as World Environment Day (WED). Under the slogan “Only One Earth”, the first celebration occurred in 1974. Environmental awareness, environmental concern, environmental attitude, and environmental behaviour of individuals, as well as their academic background, are potential factors that may help in coping with the climatic challenges the world is currently experiencing (Wu et al. 2023). However, the level of awareness about environmental adversaries varies as age, experience, and income levels vary (Maria et al. 2022), whereas the attractiveness of individuals toward climate issues is based on their awareness (Urbańska et al. 2021). For example, the examined results of a study in Indonesia revealed that those respondents who were fully aware of environmental threats openly suggested stopping the causes of environmental degradation (Kalalo 2019). This evidence shows that environmental consciousness converts normal anthropogenic activities into sustainable practices such as green consumption, lobbying for readjusting corporate business practices to include sustainability efforts (Barrón et al. 2022). Lack of awareness regarding environmental concerns not only hinders the achievable environmental targets but also detracts from the quality of life in a country. Eventually, a substandard climate at the national level may delay climate action globally.

Information and communication technology tools for social applications have changed the methods of obtaining and sharing knowledge related to environmental concerns among environmental activists (Knupfer et al. 2023). In this regard, digitalization has made it convenient to acquire various knowledge in just one click. Therefore, digital communication methods can effectively increase public awareness about environmental challenges and promote climate action measures (Tzeremes et al. 2023). The Internet and mobile cellular subscription usage reflects a country’s level of connectivity with its citizens and the rest of the world. The mentioned channels enable populations to keep themselves aware of environmental issues (Y. Wang 2014). International environmental organizations also leverage social media platforms to promote environmental awareness (Kaur and Chahal 2018). Citizens can be educated about environmental concerns inside and outside the classroom (Ardoin et al. 2020). It has been observed that an increase in higher education has a mitigating effect on climate change (Eyuboglu and Uzar 2021). This is because education leads to better awareness and understanding of environmental issues and increases the ability to develop and implement sustainable practices (Ogunbode and Arnold 2012). In light of the discussion, the current study also advocates the significance of the fundamentals of acquiring knowledge as an effective tool of disseminating environmental awareness. Hence, for examining the moderating impact of environmental awareness, this study structures environmental awareness index (*EAI*), comprising individuals using the Internet, fixed line and cellular phone subscriptions, and government expenditures on education.

Literature shows abundant work regarding the influence of economic growth and population-related factors on environmental degradation. Some studies presented that offering loans on favourable terms for environmentally friendly projects could improve environmental quality. On the contrary, a few studies underscored the trade-off, indicating that factors promoting economic growth often led to environmental degradation. For instance, energy, trade, and FDI foster economic growth but simultaneously degrade ecological quality. The literature on monetary aspects reveals notable gaps, particularly concerning specific factors like regional variations, income levels, and temporal dynamics. Recognizing these gaps, the current study aims to contribute by investigating the influence of monetary growth-associated variables on environmental quality in Pakistan. The current study fills the gaps by utilizing the latest datasets and methodologies, which offers a more comprehensive understanding of the subject matter.

Moreover, the discussed literature revealed that demographic sprawl leads to encroachment on natural habitats, disrupting ecosystem functionality. The existing studies focus on specific regions. Hence, their findings may not be universally applicable, and regional factors could introduce bias. In the demographic context, the overarching theme suggests that population growth is generally associated with negative environmental impacts. Hence, it underscores the significant connection between demographic aspects and ecological outcomes. It is crucial to acknowledge the presence of regional biases. In this context, the varying outcomes exhibit the research gap from regional, temporal, and methodological extents. Hence, the current study fills these gaps as well.

Furthermore, the existing studies discussed the role of information and communication technology in disseminating environmental awareness. Related literature also suggests a positive correlation between education and environmental awareness, but simultaneously exhibits the importance of individual values, social norms, and other factors. The awareness strategies may vary across cultures, and what works in one country or region may not have the same impact elsewhere. For instance, existing studies focusing only on information and communication technology (ICT) tools for promoting awareness may overlook the digital divide. These studies do not fully capture the current dynamics of technology’s role in environmental awareness due to the rapid evolution of technology and societal changes. For example, Pakistan has 87.35 million active Internet users and 191.8 million cellular connections.^9^ Previous studies have also emphasized the significance of formal education in keeping citizens aware of environmental concerns. Given that, the current study investigates the moderating influence of Internet use, telephone, and formal education by estimating the environmental awareness index (*EAI*).

## Theoretical framework

A few existing theories support the nexuses among the variables of concern. The limits to growth (LTG) theory affirmed the interconnectedness of physical growth and extreme weather exposures (Meadows et al. 1972). The LTG introduced the concept of “degrowth” to address environmental issues. However, halting growth received criticism because the degrowth strategy was neither convincing contemporarily nor currently (Alexander et al. 2018). Besides that, a book entitled “An Essay on the Principle of Population” by Thomas Robert Malthus in 1798 showed that populations grow exponentially, whereas resources do not (Malthus 1960). As a result of overexploitation of resources, growing populations contribute to environmental degradation. Malthus suggested preventive and positive checks strategies for controlling population growth, known as the Malthusian trap. The preventive checks involve birth control and delayed marriages. At the same time, the positive check praised the natural and social causes of the decreasing population, such as wars, diseases, and malnutrition (Glass and Appleman 1976). However, the theory exhibits demographic aspects as significant drivers of environmental degradation but has limitations. It assumes a linear relationship between population growth and resource scarcity, overlooking the potential for technological advancements, changes in consumption patterns, and innovative solutions such as environmental awareness among the populations that may alter this relationship. The Malthus theory supports our assertion that demographic aspects could influence the environment.

Furthermore, the environmental Kuznets curve (EKC) philosophy argues that economic growth escalates environmental degradation at the beginning, but after reaching a certain level, it eventually reverses the hazardous effects of growth on the atmosphere (Murshed et al. 2021). However, it is uncertain if the environment will improve (Ozcan et al. 2020). We encountered several factual uncertainties as we pondered the idea that economic growth could remedy environmental degradation. *Firstly*, it remains unclear how much economic growth would be necessary to address environmental degradation. *Secondly*, it is ambiguous how many resources would be consumed in the pursuit of economic growth to address environmental problems, potentially exacerbating the very issue we seek to solve. *Thirdly*, the EKC hypothesis, which postulates an inverted U-shaped relationship between income and environmental degradation, often includes the square of income as a variable in many studies. However, the proxy used for ecological degradation is typically kept at the same value, even though income and environmental degradation are expected to increase simultaneously due to the link between economic growth and environmental degradation. Even if we ignore this reason, it still raises an ethical question: would it be natural justice to destroy the environment until and unless countries become richer for solving their environmental issues? No, it is not because, in just a few decades, anthropogenic activities have increased temperatures and sea levels to the degrees usually expected to be reached in centuries (Cimato and Mullan 2010).

The stated concerns emphasize that economic growth alone cannot reverse environmental degradation. Hence, the current study presents a novel approach to consider, which emphasizes utilizing existing channels of acquiring knowledge for disseminating environmental awareness to curb, control, and mitigate environmental degradation. Our motto for making peace with nature is to make citizens aware of environmental concerns rather than waiting to become rich first. Because environmental degradation is an immediate threat worsening with each passing day, nature requires urgent action.

**Fig. 4:**
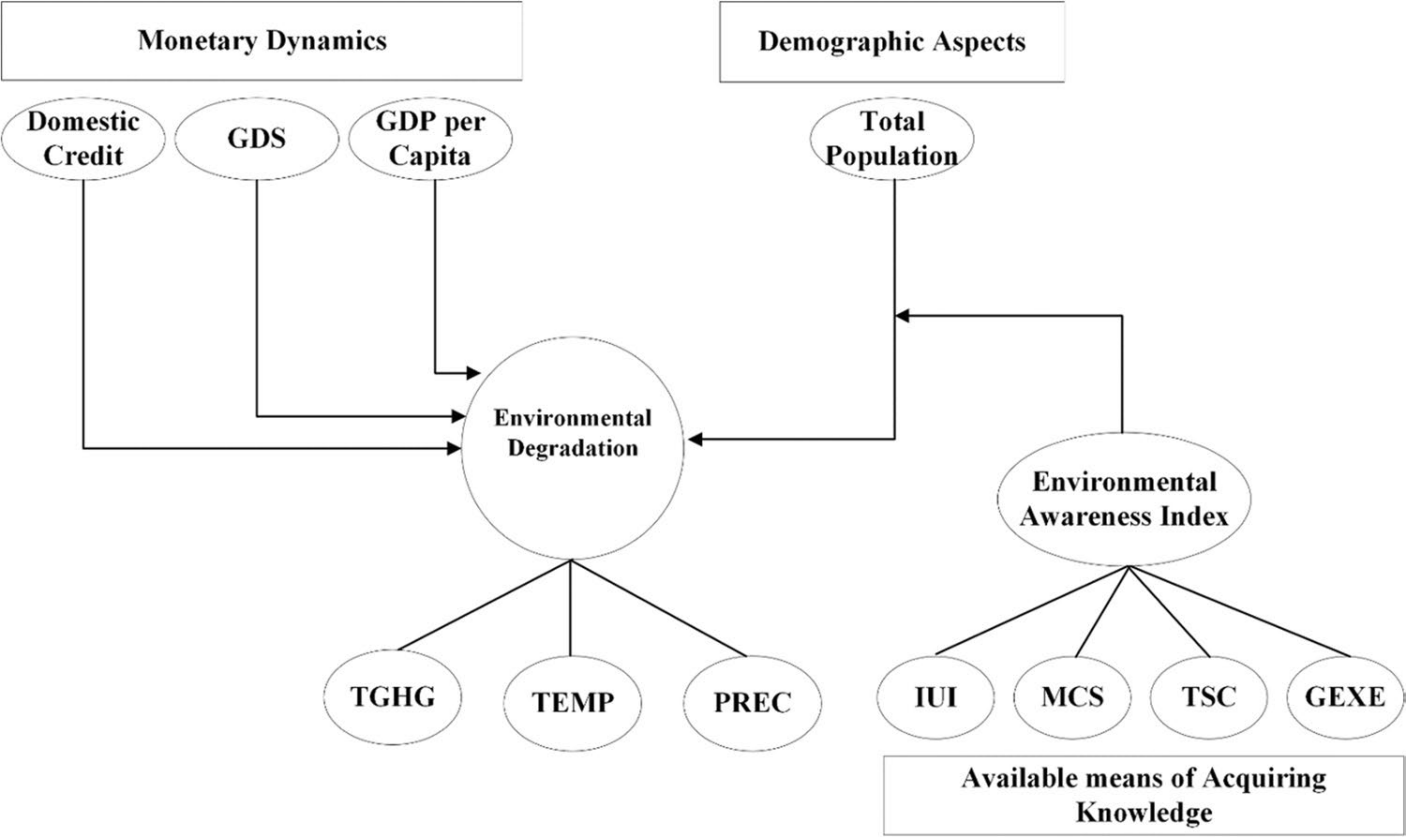
Theoretical framework

In light of the above discussion, it is expected that the basics of acquiring environmental awareness influence populations to voluntarily avoid anthropogenic activities that degrade the environment, such as deforestation, overfishing, burning garbage, agriculture residue, and medical waste in the open air. For validation, this study constructs an environmental awareness index (*EAI*) comprising indicators such as individuals using the Internet (IUI), mobile cellular subscriptions (MCS), fixed telephone subscriptions (TSC), and government expenditure on education (GEXE). The study then multiplies the estimated *EAI* by the total population (*TOP*), creating an interaction term. This approach provides valuable insights into how both unaware and environmentally aware populations influence the environment in Pakistan. To broaden the understanding of environmental degradation, the study includes the environmental degradation index (*EDIN*), which incorporates total greenhouse gas emissions (TGHG), temperature (TEMP), and precipitation (PREC). The study’s conceptual framework is structured in Fig. 4.

## Material and methods

### Data collection and measurement units

The time-series data from 1995 to 2019 has been collected for the variables of interest. The study examines the impact of GDP per capita, domestic credit, savings, total population, and environmentally aware population on environmental degradation in Pakistan. Environmental degradation can be quantified through indicators such as CO_2_, CH_4_ sources, N_2_O sources, and F-gases, including HFCs, PFCs, and SF6 (Razzaq et al. 2023). Rainfall is also an essential climatic factor determining areas at risk of land degradation and possible desertification (“World Meteorological Organization” 1963). Furthermore, increasing temperature causes melting glaciers and rising sea levels and triggers abrupt precipitations (U.S. Global Change Research Program 2018). Considering the significance of underlined measures of the environment, the current study frames the environmental degradation index (*EDIN*). A wide range of economic growth-related indicators can be used to track the monetary progress (Banco Mundial 2019). Accordingly, this study utilizes GDP per capita, domestic credit to the private sector, and gross domestic savings as monetary growth measures. The study also uses one of the demographic aspects: total population, which is the de facto definition of population.

The current study advocates that the fundamentals of acquiring knowledge help populations remain aware of environmental degradation. In this regard, formal education and communication channels are statically significant sources of environmental awareness. The individuals using the Internet and telephone reflect how people of a particular country are in touch with fellow citizens and the world. Every local and international environmental organization uses social media platforms to disseminate environmental awareness. Fortunately, people around the globe are active users of social media. With the help of such platforms, ordinary people can also monitor the air, water, and climate quality they are surrounded by (Mallick and Bajpai 2019). It is statistically proven that people using the Internet and having mobile phones are more environmentally aware than people not using such platforms (Ogunbode and Arnold 2012). The list of variables, respective descriptions, acronyms/symbols, the hypothetical impact of the variables on environmental degradation, and sources of data are illustrated in Table 1.

**Table 1:** List of variables

Variables	Symbol	Notation	Hypothetical impact	Data sources
**Dependent variables**				
Environmental degradation index	*EDIN*	Environmental degradation	N/A	Created by authors by using the PCA technique
**Independent variables**				
GDP per capita	ln*INC*	Monetary development	+	WDI
Domestic credit to the private sector by banks	ln*DCPS*	Monetary development	+	
Gross domestics savings	ln*GDS*	Monetary development	+ / −	
Population, total	ln*TOP*	Demographic trends	+	
Environmental Awareness Index	*EAI*	Environmental awareness	N/A	Created by authors
The interaction term (ln*TOP* × *EAI*)	*EATP*	Environmentally aware population	-	
**The environmental degradation index comprises**				
Total greenhouse gas emissions were taken as kilotons of CO_2_ equivalent			TGHG	WDI
The temperature was taken as the annual average temperature in Celsius			TEMP	CCKP
Precipitation was taken as annual precipitation in millimeters			PREC	
**The environmental awareness index comprises**				
Individuals using the Internet were taken as a percentage of the total population			IUI	WDI
Mobile cellular subscriptions are taken per 100 persons			MCS	
Fixed telephone subscriptions are taken per 100 persons			TSC	
The government’s total expenditure on education is measured as a ratio to the GDP			GEXE	

### Data treatment

The datasets are collected from diverse sources; hence, the measurement units of gathered data are also different. Therefore, a natural logarithm (ln) is calculated for the variables not used in estimating indexes. Meanwhile, the min–max normalization technique is applied to the variables used in composite indexes (Renzhi and Baek 2020).

1 $${V}_{n}=\left(\frac{\genfrac{}{}{0pt}{}{0}{{X}_{o}-{X}_{{\text{min}}}}}{\genfrac{}{}{0pt}{}{{X}_{{\text{max}}}-{X}_{{\text{min}}}}{1}}\right)$$

where ${V}_{n}$ indicates the normalized value of each attribute, ${X}_{o}$ shows the original quantity of characteristics, ${X}_{{\text{min}}}$ refers to the smallest number of the original attribute, and ${X}_{{\text{max}}}$ is the maximum value of the original attribute.

**Fig. 5:**
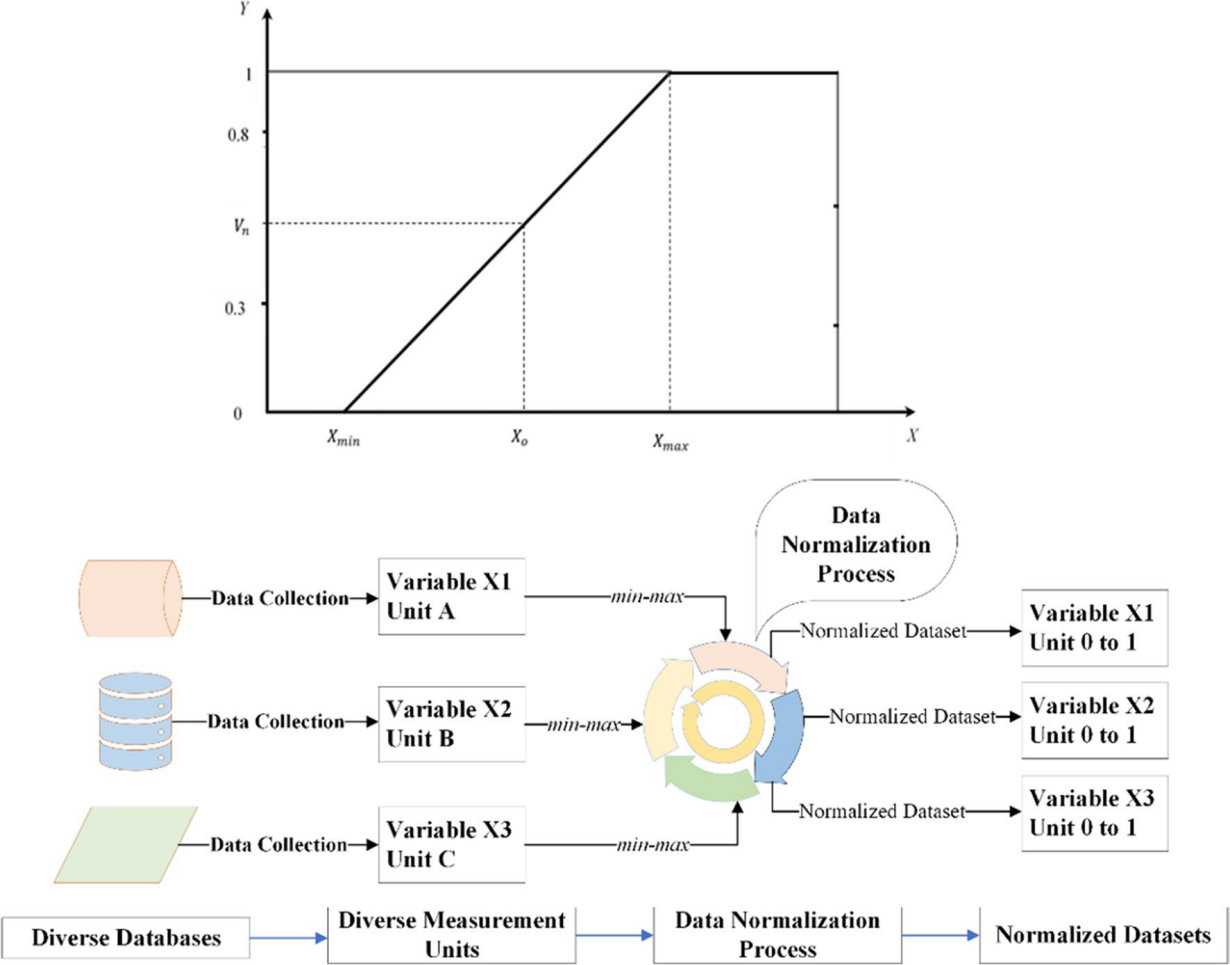
Min–max illustration

Figure 5 portrays the min–max normalization function. On the *x*-axis of the first diagram*,* the original datasets contain any value in the range of ${X}_{{\text{min}}}\mathrm{ to }{X}_{{\text{max}}}$ and after employing data normalization methodology, ${V}_{n}$ is obtained at any point on the *y*-axis ranging from 0 to 1. The second diagram elaborates on the concept, which shows that measurement units of the datasets are transformed into the range of zero and one.

### Principal component analysis (PCA)

The environmental degradation index (*EDIN*) and environmental awareness index (*EAI*) are calculated using the principal component analysis (PCA) technique. The technique obtains good-fit components for a system of points (Pearson 1901). This method reduces the dimensionality of large datasets and enhances data interpretability by focusing on key variables in original datasets (Hotelling 1933). The required indices are calculated as follows (AlShiab et al. 2020).

2 $$X = \left[\begin{array}{cccc}{X}_{\mathrm{1,1}}& {X}_{\mathrm{1,2}}& \dots & {X}_{1,p}\\ {X}_{\mathrm{2,1}}& {X}_{\mathrm{2,2}}& \dots & {X}_{2,p}\\ \vdots & \vdots & \cdots & \vdots \\ {X}_{n,1}& {X}_{n,2}& \cdots & {X}_{n,p}\end{array}\right]$$

where *X*s are individual experiments of *p* variables for *n* number of observations.

This technique standardizes the variables by eliminating components with smaller contributions to the system. PCA usually generates various principal components (*PC*_1_, *PC*_2_….*PC*_*n*_), where the first generated component enlightens a higher percentage of the total variance than the others. The linear combination of *X*_1_ to *X*_p_ in *PC*_1_ is as follows.

3 $${PC}_{1}={a}_{1}{X}_{1}+ {a}_{2}{X}_{2}+\dots +{a}_{p}{X}_{p}$$

The first component is obtained by multiplying the data matrix into variable loadings as follows.

4 $${PC}_{1}=X\times V$$

where *PC* principal components, *X* data matrix, and *V* loadings.

**Fig. 6:**
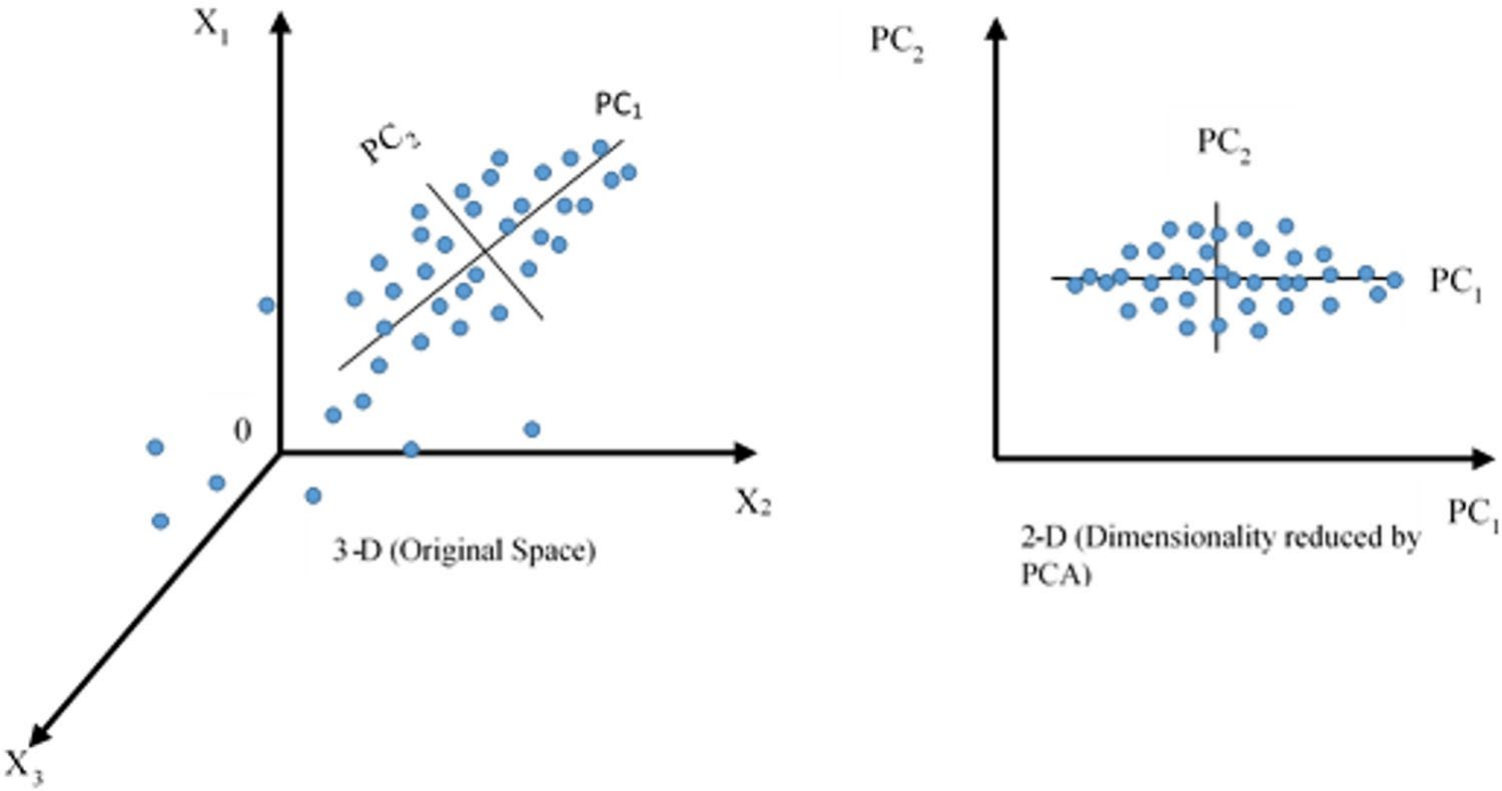
Principal components at sample space (Loukas 2020)

Furthermore, the orthogonal transformation replaces the large sets of correlated variables with the best fit smaller sets of uncorrelated variables in a system of points space as exemplified in Fig. 6.

### Unit root tests

Before moving to advanced econometric techniques, the variables are analysed for order of integration (Raihan 2023). It is crucial to know whether the variables are stationary at level, first difference, or both (Voumik et al. 2023). Therefore, the current study employs augmented Dickey-Fuller (ADF) and the Phillips-Perron (PP) unit root tests (Taneja et al. 2023). The mentioned stationarity tests help to ensure that none of the variables is cointegrated at the second difference. For each variable, these tests examine the presence of the unit root of ${y}_{t}$ in Eqs. 5 and 6. The Phillips-Perron unit root test is a non-parametric substitute for the ADF test, which deals with the data’s serial correlation and heteroskedasticity issue (Okunlola et al. 2019). Moreover, the current study also applies the KPSS (Kwiatkowski et al. 1992) unit root test. The mathematical forms of the ADF and PP unit root tests are illustrated as Eqs. 5 and 6, respectively.

5 $${\Delta y}_{t}=cn+\partial t+{\delta y}_{t-1}+\sum_{j=1}^{n}{\vartheta }_{j}{\Delta y}_{t-j}+{\in }_{t}$$

6 $${\Delta y}_{t}=\theta +\sum_{j=1}^{n}{\gamma }_{j}{\Delta y}_{t-1}+ {\varepsilon }_{t}$$

H_0_:$$\partial =0$$

H_1_: $$\partial \ne 0$$

In the above equations, ${\Delta y}_{t}$ and ${\Delta y}_{t-1}$ show first differences of the datasets, *cn* and $\theta$ are constants, $\partial$ is the coefficient on the trend at time *t*, $\delta$ is the coefficient of lagged values at *n*, $\sum_{j=1}^{n}{\vartheta }_{j}{\Delta y}_{t-1}$ and $\sum_{j=1}^{n}{\gamma }_{j}{\Delta y}_{t-1}$ present summations of lagged at first difference, and ${\in }_{t}$ and ${\varepsilon }_{t}$ are residuals.

### Econometric modeling

The current study frames the raw form of the mathematical as Eq. 7. The equation helps to determine and construct the basic econometric model of the study on which long-run and short-estimation models are based (Nondo and Kahsai 2020).

7 $$EDIN = f (INC, DCPS, GDS, TOP, TOP \times EAI)$$

where *EDIN* is for environmental degradation, *INC* shows the GDP per capita, and *DCPS* represents domestic credit to the private sector by banks. *GDS* represents gross domestic savings, *TOP* signifies the total population, whereas *TOP* × *EAI* is an interaction term for an environmentally aware population.

Using the above equation, we construct the basic econometric models as follows.

#### Basic model

8 $${EDIN}_{it}={\vartheta }_{0}+{\vartheta }_{1}{EDIN}_{it-1}+{\vartheta }_{2}{{\text{ln}}INC}_{it}+{\vartheta }_{3}{{\text{ln}}DCPS}_{it}+{\vartheta }_{4}{{\text{ln}}GDS}_{it}+{\vartheta }_{5}{{\text{ln}}TOP}_{it}+{\vartheta }_{6}{EATP}_{it}+{\tau }_{i}+{\delta }_{it}$$

where ln shows the natural logarithms of variables, *EDIN* is the environmental degradation index, ln*INC* is used for gross domestic product per capita, ln*DCPS* shows domestic credit to the private sector by the banks, ln*GDS* displays gross domestic savings, ln*TOP* indicates total population, and *EATP* is interaction terms of the total population and environmental awareness index. Furthermore, ${\tau }_{i}$ represents country-specific properties, *i* is for country, and *t* demonstrates time, and ${\delta }_{it}$ is the error term.

### ARDL bounds testing technique

After testing unit roots, the next step is determining whether the long-run association among the regressors exists. The study applies Pesaran’s autoregressive distributed lag (ARDL) bound testing approach to determine the long-run co-existence among the variables (Pesaran et al. 2001). The method provides asymptotic critical values of the lower and upper bound at different significance levels (Dogan and Turkekul 2016; Forgenie and Khoiriyah 2023; Oroud et al. 2023). Numerous other techniques, such as those of Johansen and Juselius (1990), Johansen (1988), and Engle and Granger (1987), can also be used for the same purpose. However, the ARDL method is comparatively advantageous (Sam et al. 2019). For example, the ARDL method does not limit variables to having the same integration order (Raihan et al. 2023). Instead, it produces robust outcomes even if the variables are stationary at level I(0) or first difference I(1) or have a mixed order of integration (Sunde et al. 2023). Moreover, the ARDL technique examines variables at different lag lengths, recognizing that the relationship between variables may not have the same lag structure. The method also explores relationships among the variables in a single equation, whereas traditional cointegration techniques use multiple equations to capture the interdependencies among variables (Ozturk and Acaravci 2011). The highlighted reasons make the ARDL model advantageous because it has a flexible specification and computational efficiency (Hao 2023). In the first step, the ARDL technique estimates the long-run cointegration among the variables, whereas in the next stage, it estimates long-run coefficients (Bhattacharjee and Das 2023; Bunnag 2023). After establishing long-run relations among the variables, this study transforms the raw form of mathematical equations into the standard form of the log-linear models. The model estimates the short-run and long-run impacts of GDP per capita, domestic credit, savings, total population, and environmentally aware population on environmental degradation. The minute cointegration is confirmed, and the model specifies an error correction form (Aluwani 2023). That combines short-run coefficients and long-run adjustments in the single error correction model (*ECM*), which helps to analyse both long-run and short-run parameters simultaneously(Mohammed Idris et al. 2023). The lag length can be selected by the Akaike information criterion (AIC) and other criteria (Bunnag 2023). The mathematical equations of the models are calculated as follows.

#### Cointegration model

9 $$\begin{array}{l}\text{ln}DV=\partial_0+\partial_1{\text{ln}DV}_{t-1}+\partial_2{\text{ln}IVone}_{t-1}+\partial_3{\text{ln}IVtwo}_{t-1}\\\;\;\;\;\;\;\;\;\;\;+\dots+\partial_n{\text{ln}IVn}_{t-1}+\sum\limits_{j=1}^n\partial_5{\Delta\text{ln}DV}_{t-j}+\sum\limits_{j=1}^n\partial_6{\Delta\text{ln}IV}_{t-j}\\\;\;\;\;\;\;\;\;\;\;+\dots+\sum\limits_{j=1}^n\partial_n{\Delta\text{ln}IVn}_{t-j}+\varepsilon_{it}\end{array}$$

$$\begin{array}{l}{\mathrm H}_0:\;\partial_1=\partial_2=\partial_3=\dots\partial_{\mathrm n}=0\\{\mathrm H}_1:\partial_1\neq\partial_2\neq\partial_3\neq\dots\partial_n\neq0\end{array}$$

where *DV* represents the dependent variables, *IV* shows the independent variables, ${\varepsilon }_{it}$ shows residuals, *t* is for time, and *i* is used as a country-specific subscript. The null hypothesis suggests no long-run cointegration among the regressors.

#### Long-run model


10


#### Short-run model


11


where *ECM* shows the error correction of the short-run coefficients, which reflects the adjustment speed of the coefficients into long-run cointegration, and ${\varepsilon }_{it}$ is the error term. Furthermore, the stability and reliability of the models are checked by CUSUM and CUSUMSQ as well as through residual diagnostics and Ramsey RESET (Gwachha 2023; Ojaghlou et al. 2023; Razmi and Razmi 2023).

## Results and discussions

### PCA results of environmental degradation (EDIN) and awareness (EAI)

Principal component analysis (PCA) estimations of the environmental degradation index (*EDIN*) are depicted in Table 2. PCA outcomes show that principal component one (*PC*_1_) has the highest eigenvalue of 1.641. The eigenvalue is a factor by which a principal component is scaled. Moreover, *PC*_1_ also explains a higher percentage of variance, 54.70%, compared to other variables in the original dataset. Since *PC*_1_ reasonably shows the highest rate of variance and possesses the highest eigenvalue compared to *PC*_2_ and *PC*_3_, the first *PC* is accepted as *EDIN* for measuring environmental degradation in Pakistan. An orthogonal rotation is applied to minimize the number of variables based on higher loading on each element. Thus, the interpretation of factors was shortened. Loading values of the estimated index (*PC*_1_) range from − 0.037 to 92.20%, determining that *PC*_1_ is correlated with total greenhouse gas emissions, annual average temperature, and annual mean precipitation at 92.20%, 85.70%, and − 3.70%, respectively. The factor loadings indicate the influence of each characteristic on an extracted principal component in rotated space.

**Table 2:** PCA of environmental degradation index

Components	Eigenvalue	Difference	Variance (%)	Cumulative variance (%)		
*C*_1_	1.641	0.588	54.701	54.701		
*C*_2_	1.053	0.746	35.084	89.786		
*C*_3_	0.306		10.210	100.00		
Indicators	*PC*_1_	Kaiser–Meyer–Olkin		Bartlett’s test		
				Chi-square	df	Sig
TGHG	0.922	0.393		14.103	3	0.003
TEMP	0.857					
PREC	− 0.037					

**Fig. 7:**
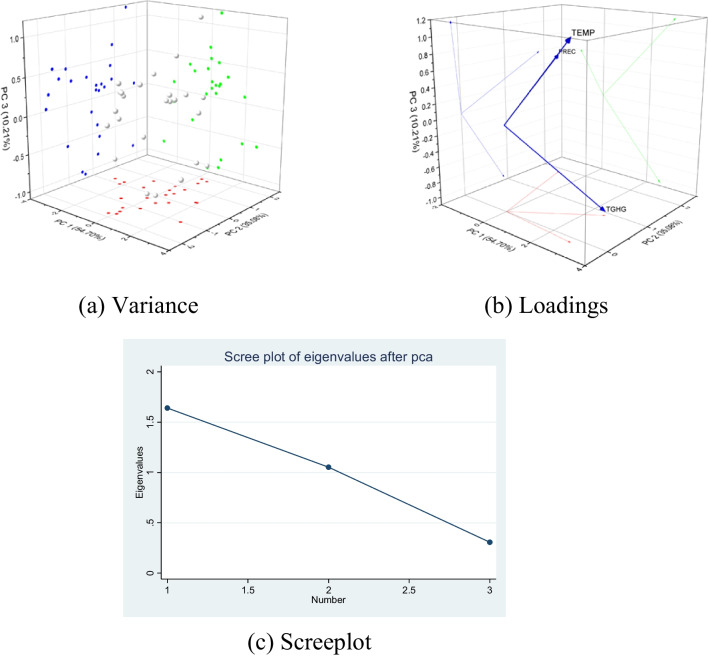
PCA outcomes of *EDIN*

Moreover, selecting the best-fit *PC* as an index is based on its eigenvalue and percentage of variance. Consequently, the remaining two components are skipped, assuming the environmental degradation index score. Kaiser–Meyer–Olkin (KMO) result shows the sampling competence with the value of 0.393, and Bartlett’s test of sphericity is valid at a *p*-value of 0.003 with X2 14.103. Figure 7a displays the percentage of the variance of the components, whereas Fig. 7b illustrates the loadings of principle components in three dimensions. Figure 7c shows the eigenvalues of the index.

The PCA results of the environmental awareness index (*EAI*) in Table 3 illustrate that *PC*_1_ has the highest eigenvalues of 2.063. Furthermore, the first *PC* explains 51.580% of the variance in the dataset of environmental awareness, which is comparatively higher than the other components. Hence, based on the eigenvalue and percentage of the variance of the awareness indicators, this study accepts principal component one as an index value of environmental awareness. Orthogonal varimax rotation reduced the interpretation of awareness aspects. Factor loading of the accepted component ranges from 5.30 to 97.7%, meaning that the environmental awareness index has 97.70%, 96%, 21.90, and 5.30% influence on cellular subscriptions, Internet use, government’s educational expenditure, and fixed telephone line subscriptions, respectively.

**Table 3:** PCA of environmental awareness index

Components	Eigenvalue	Difference	Variance (%)	Cumulative variance (%)		
*C*_1_	2.063	0.764	51.580	51.580		
*C*_2_	1.299	0.724	32.472	84.052		
*C*_3_	0.575	0.511	14.370	98.417		
*C*_4_	0.063		1.580	100.00		
Indicators	PC_1_	Kaiser–Meyer–Olkin		Bartlett’s test		
				Chi-square	df	Sig
IUI	0.960	0.348		50.824	6	0.000
MCS	0.977					
TSC	0.053					
GEXE	0.219					

**Fig. 8:**
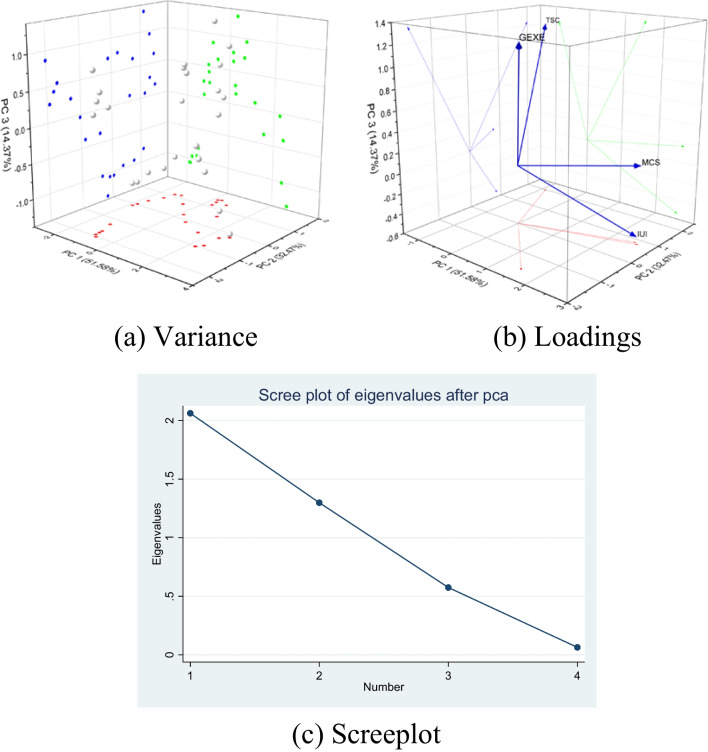
PCA outcomes of *EAI*

Moreover, KMO results are 0.348, and the test of sphericity is valid at a 0.000 significance level with 50.824 chi-square. Figure 8a displays the percentage of the variance of the components, whereas Fig. 8b illustrates the loadings of principle components in three dimensions. Figure 8c shows the eigenvalues of the index.

### Unit root test results

The results of augmented Dickey-Fuller (ADF), Phillips-Perron (PP), and KPSS (1992) unit-root tests are illustrated in Tables 4, 5, and 6, respectively. The ADF and PP tests are conducted on the exogenous parameters of none, constant, and both constant and linear trends. While the KPSS has only two exogenous measurement parameters in this regard, we analysed the results of the KPSS on constant and both constant and linear trends. Testing unit roots in the data sets enables us to decide the order of integration in the datasets. In this regard, we have designed a statistical strategy to analyse any evidence of stationarity in horizontal and vertical datasets. Horizontal strategy in this regard means that if there is any evidence of stationarity in data observed at none, constant, or at the constant and linear trend, it will reject the null, suggesting “no stationarity”. Vertical integration decisions are based on the stationarity of variables vertically. If there is any evidence of stationarity in the data at any exogenous vertical, it would help in deciding the order of integration. The findings of all the employed unit-root tests for variables of concern are cointegrated at the level and first difference. Hence, the integration decision for all the variables is determined to be a mixed order of integration.

**Table 4:** Augmented Dickey-Fuller Test Results

*t*-Stat		Level			First Dif		Decision
	N	C	CT	N	C	CT	
*EDIN*	− 1.409	− 1.366	− 3.925**	− 6.413*	− 6.848*	− 3.765**	Mixed
ln*INC*	1.541	0.118	− 4.251**	− 1.654***	− 3.608**	− 3.455***	Mixed
ln*DCPS*	− 0.996	− 1.107	− 2.293	− 3.588*	− 3.642**	− 3.572**	I(1)
ln*GDS*	− 1.462	− 0.239	− 2.349	− 5.647*	− 6.096*	− 6.296*	I(1)
ln*TOP*	1.193	− 4.437*	− 1.429	− 3.258*	− 2.057	− 8.129*	Mixed
*EATP*	− 0.296	0.032	− 3.485***	− 1.363	− 2.675***	− 2.675	Mixed

ADF conditions: *MacKinnon (1996) one-sided *p*-values, Lag length automatic selection at Akaike information criterion (AIC)

*Dif.* difference, *N* exogenous none, *C* exogenous constant, *CT* exogenous constant, linear trend

*, **, and *** show significance at 1%, 5%, and 10%, respectively

**Table 5:** Phillips-Perron (PP) test results

Adj. *t*-stat		Level			First Dif		Decision
	N	C	CT	N	C	CT	
*EDIN*	− 1.591	− 1.451	− 3.921**	− 6.413*	− 9.027*	− 9.023*	Mixed
ln*INC*	3.402	0.998	− 1.928	− 1.670***	− 2.338	− 2.562	I(1)
ln*DCPS*	− 0.868	− 0.998	− 1.832	− 3.572*	− 3.630*	− 3.561**	I(1)
ln*GDS*	− 1.462	− 0.239	− 2.240	− 5.609*	− 6.090*	− 6.296*	I(1)
ln*TOP*	9.256	− 8.936*	− 0.895	− 1.957**	− 1.213	− 1.574	Mixed
*EATP*	− 0.332	0.412	− 2.098	− 1.288	− 2.580	− 2.600	-

PP conditions: bandwidth selection: Newey-West automatic and spectral estimation method is Bartlett kernel

*Dif.* difference, *N* exogenous none, *C* exogenous constant, *CT* exogenous constant, linear trend

*, **, and *** show significance at 1%, 5%, and 10%, respectively

**Table 6:** Kwiatkowski-Phillips-Schmidt-Shin (KPSS) test results

H_0_: Variable is stationary							
LM-stat	Level		First Dif			Asymptotic critical values^T^	
	C	CT	C	CT	Decision	Constant	
*EDIN*	0.691	0.088*	0.266*	0.235	Mixed	1% level	0.739000
ln*INC*	0.705	0.097*	0.239*	0.069*	I(1)	5% level	0.463000
ln*DCPS*	0.466	0.117*	0.099*	0.073*	I(1)	10% level	0.347000
ln*GDS*	0.668	0.108*	0.117*	0.061*	I(1)	Constant and trend	
ln*TOP*	0.729	0.193**	0.637**	0.053*	I(1)	1% level	0.216000
*EATP*	0.713	0.089*	0.163*	0.125**	Mixed	5% level	0.146000
						10% level	0.119000

*N* exogenous none, *C* exogenous constant, *CT* exogenous constant, linear trend

*, **, and *** show significance at 1%, 5%, and 10%, respectively

^T^Kwiatkowski et al. (1992, Table 1)

### Cointegration results

Cointegration helps to underline the long-run association among the variables. The current study conducts a bound testing approach to examine the long-run relationships among the factors under study. The null hypothesis suggests that no long-run connection exists between the elements. The test outcomes are evaluated with the lower bound critical value of 5%. If *F*-statistics values are lower than 5%, we cannot reject the H_0_. Table 7 exhibits the results of long-run associations. Results reveal that the null hypothesis is rejected at a 1% significance level. In light of the statistical outcomes, all the variables have long-run associations.

**Table 7:** Bounds test results

H0: No long-run relationships among the variables				
AIC-based ARDL model (1, 2, 0, 0, 0, 0)				
Test statistic		Significance	Critical values	
*F*-statistic	Regressors (K)		Lower bound	Upper bound
5.456*	5	10%	2.26	3.35
		5%	2.62	3.79
		2.5%	2.96	4.18
		1%	3.41	4.68

*shows significance at 1%

### Long-run and short-run findings

Long-run and short-run results of the ARDL model are reported in Table 8. Estimated outcomes assert the influence of independent variables on the dependent variable in the long and short runs. The findings reveal that a one-unit increase in the GDP per capita causes 10.982% of environmental degradation in Pakistan. Since the environmental degradation in this study is a composite index of total greenhouse gas emissions (TGHG), temperature (TEMP), and precipitation (PREC), the results articulate that an increase per capita in Pakistan escalates greenhouse gas emissions, increases the country’s annual mean temperature and causes the abrupt waterfall from the sky. Increasing real income that is detrimental to environmental quality over a longer period was also discovered by Dogan and Turkekul (2016) and Ertugrul et al. (2016). In the short run, the study finds that per capita income is favourable for the environment in Pakistan because the values are negatively significant, showing a numerical outcome of − 19.031. Domestic credit to the private sector is also positively significant to the environmental degradation index over long and short periods. Numerical outcomes reveal that in Pakistan, when banks disperse 1% of the loan to the private sector, it causes 1.122% and 1.193% environmental degradations in the long run and the short run, respectively.

**Table 8:** Long-run and short-run estimations

AIC-based ARDL model (1, 2, 0, 0, 0, 0)						
Dependent variable: *EDIN*						
***Long-run statistics***		**ln*INC***	**ln*DCPS***	**ln*GDS***	**ln*TOP***	***EATP***
Coefficient		10.982*	1.122***	1.196	10.267**	− 0.076***
*t*-statistics		3.490	1.911	1.512	2.376	− 1.853
Probability		0.004	0.077	0.153	0.032	0.085
***Short-run statistics***	***C***	**ln*INC***	**ln*DCPS***	**ln*GDS***	**ln*TOP***	***EATP***
Coefficient	− 295.4**	− 19.031**	1.193***	1.271	10.911***	− 0.080
*t*-statistics	− 2.588	− 2.772	1.850	1.601	2.005	− 1.670
Probability	0.022	0.015	0.086	0.131	0.065	0.111
***ECM regression***	***C***	**D (ln*INC*)**	**D (ln*INC* (− 1))**		**Coint. Eq**	
Coefficient	− 295.4*	4.497	− 19.031*		− 1.062*	
*t*-statistics	− 6.662	1.080	− 4.492		− 6.665	
Probability	0.000	0.299	0.000		0.000	

*, **, and *** show significance at 1%, 5%, and 10%, respectively. Lag length automatic selection at Akaike information criterion (AIC)

Furthermore, the study reveals that gross domestic savings are positively insignificant in the long run as well as in the short run. Because *GDS* decreases the propensity of consumption, such a decline could sequentially drop the economic output, which is in favour of environmental quality. However, it is not unfavourable for economic growth as a whole, as the savings mobilize gross capital formation in the country. Further assessments reveal that the population growth of Pakistan is significantly increasing its ranking as an environmentally vulnerable country. This is because a 1% rise in population upsurges greenhouse house gases, temperature, and unpredictive precipitation by 10.267% in the long run and 10.911% in the short run. The current study advocates that existing means of acquiring knowledge can help Pakistan disseminate environmental awareness to the population. What this study hypothesized was that the statistical findings proved it. In this regard, the numerical outcomes reveal that a 1% rise in the dissemination of environmental awareness through the Internet, mobile and landline phones, as well as by increasing green educational budget not only mitigates the entire percentage of anthropogenic causes of environmental degradation but also improves environmental quality by 0.076% in the long run. However, in the short run, the results are negatively insignificant. This establishes that environmental awareness is not solely influenced by factors such as education level, age, and experience, as reported by previous studies (Sprague et al. 2021; Wu et al. 2023). However, the current research reveals that fostering environmental consciousness also depends on the passage of time.

In conclusion, the outcomes of the study evidence that GDP per capita, domestic credit to the private sector, and total population are detrimental to environmental quality in Pakistan. The findings are consistent with the studies considering the same monetary growth and population-related features (Abbas et al. 2020; Khan et al. 2020; Li et al. 2023). However, the varying magnitudes of the detrimental impact of the underlying monetary and demographic aspects on environmental degradation can be attributed to differences in methodology, time frames, and the countries under consideration. Furthermore, this study suggests that gross domestic savings are favourable for the environment in Pakistan due to the functional nature of savings as it increases the propensity to save rather than the tendency to consume and produce.

Additionally, the study discovers that the increasing population actively contributes to environmental degradation. Therefore, this study examines the role of environmental awareness as a potential climate action strategy to improve Pakistan's environmental conditions. Fortunately, environmental awareness has proved to be an effective solution to Pakistan’s worsening climate. The current study marks the discovery as an achievement and suggests that an environmentally aware population can help Pakistan get off the list of environmentally vulnerable nations. The cointegration equation is statistically significant at the 1% level, with a coefficient of − 1.06. The negative sign of the coefficient and the significance of the error correction term (*ECM*) indicate a solid long-run cointegration among the variables.

### Model diagnostics

**Fig. 9:**
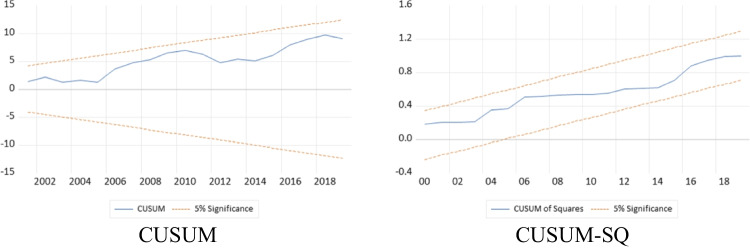
OLS-based initial stability test of the model

**Fig. 10:**
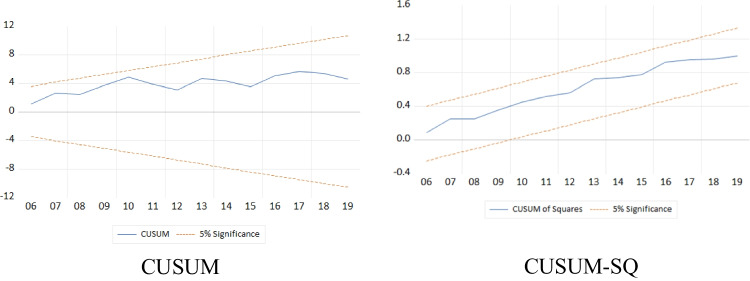
ARDL model stability

The model is examined for robustness. Figure 9 shows the initial OLS-based stability of the model. Meanwhile, Fig. 10 illustrates the stability of the ARDL bounds testing approach model. Both results reveal that the model remained stable from its inception till the end. Table 9 provides further evidence concerning the reliability of the econometric model. The results of diagnostic tests, which include the Ramsey RESET test for assessing the functional form of the regression, the Normality test for evaluating goodness of fit, the Breusch-Godfrey Serial Correlation LM test for checking autocorrelation in the errors of the regression model, and the Heteroskedasticity test for detecting heteroscedasticity in the regression model, all indicate the reliability of the model. Based on these diagnostic results, this study’s long-run and short-run findings are robust.

**Table 9:** Diagnostic results

***Ramsey RESET Test*** H_0_: Model has no omitted variables					***Jarque–Bera*** H_0_: Data is normally distributed		
		Value	df	Probability	Statistics	Prob	
*t*-statistic		0.253	13	0.804	0.921	0.631	
*F*-statistic		0.064	(1, 13)	0.804			
Likelihood ratio		0.113	1	0.737			
		***Heteroskedasticity Test:*** Breusch-Pagan-Godfrey H_0_: Homoskedasticity			***Breusch-Godfrey Serial Correlation LM Test*** H_0_: No serial correlation at up to 2 lags		
*F*-statistics		0.622			3.533		
Probability		0.747			0.062		
Chi-square		0.643			0.014		

## Conclusion and policy implications

A clean environment forms the foundation of sustainable development and human prosperity. However, it has recently been observed that environmental degradation poses a real and persistent threat to countries worldwide. The resulting impacts can be felt across the social and economic dimensions. Therefore, potential decision-making of managing environmental sustainability is inevitable in the current world order, which is shaped by the environment. In this vein, the current study provides a comprehensive understanding of monetary and demographic causes of environmental degradation and proposes environmental awareness as a panacea to environmental degradation in Pakistan. Giving importance to the Paris Agreement’s recognition of developing countries that were harshly smashed by extreme weather occurrences, the current study focused on Pakistan. The country has consistently been ranked eighth, fifth, and eighth as an environmentally vulnerable nation from 1998 to 2017, 1999 to 2018, and 2000 to 2019, respectively. Despite facing extreme weather for the past 25 years, Pakistan recently experienced a massive flood that resulted in millions of dollars in losses and displaced millions of people. The country seeks billions of dollars for rebuilding.^10^ The time series data covering the period from 1995 to 2019 was gathered to analyse the impact of GDP per capita, domestic credit to the private sector, gross domestic savings, total population, and environmentally aware population on environmental degradation.

This study took environmental degradation as the dependent variable for mathematical analysis. The independent variables included per capita real income, domestic credit, savings, total population, and environmentally aware population. Environmental degradation is represented by total greenhouse gas emissions, temperature, and precipitation. These three aspects of environmental degradation are combined into a composite index called the environmental degradation index. The environment-aware population is an interaction term calculated by multiplying the total population with the environmental awareness index (*TOP* × *EAI*). Meanwhile, the index of environmental awareness comprises individuals using the Internet, mobile cellular and fixed telephone subscriptions, and the government’s expenditures on education. To estimate the indexes, the environment and awareness-related variables are standardized to the same scale using the min–max technique before applying Karl Pearson’s principal component analysis (PCA) technique. The natural logarithms (ln) are taken for other variables of interest. To ensure robust interpretations, this study implemented a series of statistical checkpoints to rigorously assess the variables of interest at different stages of the analysis. The analysis commenced by calculating the unit root tests to determine the order of integration. The study has incorporated augmented Dickey-fuller, Phillips-Perron, and Kwiatkowski-Phillips-Schmidt-Shin unit root tests. The unit root test revealed mixed-order integration, which led to the selection of the ARDL bounds testing approach to confirm the existence of long-run cointegration among the variables. The theoretical background of the study has explained that environmental degradation is caused by monetary development and population sprawl. Likewise, this interrelationship is also statistically affirmed by the cointegration test results. After establishing the presence of long-run cointegration, the study employed ARDL short- and long-run estimation models. The study’s findings revealed that per capita income and domestic credit to the private sector harm the environment in Pakistan, whereas savings do not. Additionally, the study discovered that the increasing population actively contributes to environmental degradation. Hence, given the importance of the Paris Agreement’s 1/CP.17, Sustainable Development Goal 13, and Climate Action Act 2017 of Pakistan, this study has empirically examined the role of environmental awareness as a potential climate action strategy for improving Pakistan’s environmental conditions. Fortunately, environmental awareness has been proven to be an effective solution to Pakistan’s worsening climate situation. The findings marked this achievement and suggested that an environmentally aware population can help Pakistan get off the list of ecologically vulnerable nations. The statistical outcomes upheld the importance of the existing fundamentals of acquiring knowledge in Pakistan. The study showed that in Pakistan, publicizing environmental awareness through the existing channels of acquiring knowledge significantly converts environmentally harming anthropogenic activities into eco-friendly human activities. In this regard, the Government of Pakistan’s expenditures on education also plays a vital role. Based on empirical findings, this study proposes environmental awareness as a potential climate action strategy for making peace with nature in Pakistan.

The outcomes of this study provide empirical support to the existing legislation of the Pakistan “Climate Change Act 2017”. The study contributes to the particular areas, including (o), (p), (s), and (t) of Chapter III of Act No. F. 9(4)/2017-Legis. Based on long-run significant outcomes regarding the fundamentals of acquiring environmental knowledge, the current study establishes that environmental awareness is an immediate remedial panacea for environmental degradation in Pakistan. Therefore, Pakistani authorities may add the efficiency of their awareness programs by using existing sources of acquiring knowledge such as the Internet, mobile, and fixed telephones to inculcate climate awareness in the citizens. Also, by increasing the green education budget, the Government of Pakistan can enhance its overall climate action efforts. In this regard, the study proposes a few methods to execute the plans to enhance awareness as follows.

1. The Pakistan Ministry of Climate Change and Pakistan Environmental Protection Agency, in collaboration with the Ministry of Education and Higher Education Commission of Pakistan, designed a green education plan and enhanced the budget.
2. The Pakistan Ministry of Climate Change and the Pakistan Environmental Protection Agency, in collaboration with the Pakistan Telecommunication Authority, should take the Cellular Companies and Pakistan Telecommunication Ltd. onboard for climate awareness-related voice tunes. The Pakistani Health Ministry and the National Disaster Management Authority of Pakistan have had a brilliant experience, as they successfully motivated the citizens to take COVID-19 vaccine jabs through such messages.
3. The Pakistan Ministry of Climate Change and the Pakistan Environmental Protection Agency may enhance social media activities regarding environmental concerns.

The key innovative contributions of the study are as follows:

1. The current study extends the practical implications of Sustainable Development Goal 13 (SDG 13) by introducing environmental awareness as an effective climate action strategy in Pakistan.
2. The study also provides empirical evidence that supports the assertion of the Paris Agreement that governments can address environmental issues through legislation.
3. Similarly, the findings provide empirical support to the existing legislation of Pakistan *i.e.* the “Climate Change Act 2017”. The study contributes to the particular areas of Chapter III sub-article 8, including (o), (p), (s), and (t) of Act No. F. 9(4)/2017-Legis.
4. The current study introduces new measures of environment and awareness. Hence, the study enhances the conceptual understanding of the subject matter, broadening the findings' theoretical and mathematical significance.

The current study is limited to Pakistan only. Therefore, it is necessary to exercise caution when extrapolating the findings of this study to other countries. Additionally, a limited number of variables related to monetary growth and population aspects were utilized. Furthermore, a restricted set of variables was employed to construct environmental degradation and awareness indexes.

Future research should consider a larger sample size, as environmental threats are global issues that cannot be confined to specific countries. Moreover, future studies should also investigate numerous other monetary and demographic features. Furthermore, while our research highlights environmental awareness as a potential solution to environmental degradation, future studies may consider other factors, such as developing green finance mechanisms, including green bonds, green investment funds, environmentally sustainable projects and initiatives.

## Data Availability

All data generated or analysed during this study are included in the published article.

## Footnotes

1 https://www.worldwildlife.org/stories/3-billion-animals-harmed-by-australia-s-fires

2 https://www.washingtonpost.com/world/2021/08/11/siberia-fires-russia-climate/

3 https://www.fao.org/3/cb4894en/online/src/html/chapter-04-4.html

4 https://www.un.org/sustainabledevelopment/hunger/#:~:text=Goals%20Report%202023-,Goal%202%20Targets,sufficient%20food%20all%20year%20round.

5 https://www.unodc.org/pakistan/en/country-profile-pakistan.html

6 https://www.unesco-floods.eu/global-climate-risk-index-2021-published/

7 https://policy.asiapacificenergy.org/node/2911

8 https://www.unep.org/news-and-stories/press-release/new-unep-synthesis-provides-blueprint-urgently-solve-planetary

9 https://datareportal.com/reports/digital-2023-pakistan

10 https://www.reuters.com/world/asia-pacific/pakistan-seeks-help-with-16-bln-flood-rebuilding-un-conference-2023-01-08/
